# Taking a chance on epigenetics

**DOI:** 10.3389/fgene.2014.00205

**Published:** 2014-07-04

**Authors:** Sophie A. Lelièvre

**Affiliations:** Department of Basic Medical Sciences, Center for Cancer Research, Purdue UniversityWest Lafayette, IN, USA

**Keywords:** epigenetic modification, epigenome, primary prevention, diagnosis, risk assessment, bioethics, ancestry, chronic disease

Epigenetics has become the indispensable science for understanding the gene-environment relationships that control risk exposure, therapeutic response, and disease progression. Despite the importance of the field, not all scientists agree on how even to define epigenetics (Dupont et al., 2009); some, like me, restrict epigenetics to events that happen at the gene level, such as DNA methylation and histone modifications that constitute epigenetic marks, and the binding of proteins and RNAs involved in transcription control. Others also include miRNAs that interact with messenger RNAs. My purpose here is not to discuss semantics, but rather to consider the enormous expectations that we have placed upon epigenetics for its potential to provide quantifiable markers of risk.

The notion of risk itself is a matter of significant debate in public health. It relies on our capacity to determine when a cell or tissue has become altered to the point where a disease might have a chance of onset, recurrence, progression or resistance to treatment. Risk is also linked to statistics; individuals who display a known marker of risk are placed in an “at risk” group from which, usually, only some people will actually develop the disorder. Therefore, to address uncertainty associated with risk assessment, the individual “at risk” should be monitored, and we should prioritize the search for underlying mechanisms in order to develop prevention strategies.

Next-generation sequencing is currently the basis for large scale epigenetic analyses. But the balance between the investment of large amounts of funds in genetic sequencing and the paucity of resulting new information for cancer knowledge has raised doubt about the overall efficiency of this approach (Yaffe, 2013). With the epigenetic code consisting of more than two-dozen different epigenetic marks, the gathering of data for risk assessment would require an exponential use of such costly technologies. Nonetheless, in my opinion, epigenetics is likely to enable great strides in risk assessment because epigenetic marks are rapidly, and sometimes sustainably, modified in response to risk variations. The highly specific nature of the mechanisms that control epigenetic marks might even provide targets for strategies designed to reduce individual risk.

Indeed, our query should not be merely whether epigenetics can contribute to risk assessment, but how the information that we learn can and will be used to provide meaningful measures of risk. However, as developed in the next paragraphs, epigenetic information is unlikely to function as a sufficient single factor in risk assessment. Moreover, the epigenome that comprises epigenetic marks for all genes is development-dependent as well as tissue-dependent. Thus, a sound experimental approach is necessary to identify suitable sources of tissue for epigenetic analysis and to better understand how the epigenome would respond depending on the time period of risk exposure.

## The place of epigenetics in risk assessment

Genetic and epigenetic modifications ought to be considered together in risk investigations. Risk assessment for disease development has been conducted via screening for genetic modifications in cancers, cystic fibrosis, heart disorders, etc. Yet, the risk level can span a wide-range; for example, *BRCA1* mutation carriers have between 56 and 80% lifetime risk for breast cancer (Millot et al., 2012). Epigenetics is now considered a missing link in risk estimation that normally relies on genetics, as in the case for mood disorders (Menke et al., 2012). In these diseases, as well as in multiple sclerosis and many cancers, heritability cannot be explained only by genetic risk factors (Zhou et al., 2014). The discovery of epigenetic mechanisms involved in pathogenesis provides a new option for risk assessments. The combination of genetic and epigenetic markers confers an enhanced risk appreciation. For instance, survival from non-small cell lung cancer appears better estimated by *RGC32* methylation and *TP53* mutations together compared to mutations alone (Kim et al., 2011). These findings are encouraging. Nevertheless, making epigenetics a useful part of risk assessment will require well-developed and coordinated efforts. As pointed out by Liloglou and colleagues, for example, the sensitivity of a given assay is a major aspect in determining risk; therefore, better results might be obtained in a first approach with populations at high risk rather than the general population if the test is not highly sensitive. Also, the epigenetic field has been mainly developed via fundamental research; in order to properly utilize epigenetic marks in risk assessment, we need to develop epigenetic studies aimed at clinical biomarker discovery and validation (Liloglou et al., 2014).

Epigenetic modifications are the principal engine for changes of cell behavior and organ development (Weaver, 2009; Parfitt and Zernicka-Goetz, 2010), suggesting that it is paramount to study risk exposure in the appropriate context. For instance, epigenetic modifications have been associated with the impact of intrauterine growth restriction on lung disease via the involvement of genes, like *PPARγ*, necessary for lung development (Joss-Moore et al., 2011). Also, the real impact of epigenetic marks on the onset of toxicant-induced risk is still unclear (Alyea et al., 2012); improved knowledge of the role of epigenetic modifications on normal cell function should further this understanding. Overall, epigenetic research on non-diseased tissues is lacking. The National Institutes of Health Epigenomics Mapping Consortium may help fill part of the gap by promoting the gathering of epigenomic information from stem cells as well as primary *ex vivo* tissues representing normal conditions (Bernstein et al., 2010); still few studies make use of functional models of normalcy, especially for human tissues. A serious effort should be devoted to building and implementing models via, for instance, the design of organs-on-chips for laboratory use (Vidi et al., 2013). Moreover, because the epigenetic code is specific to each tissue type, we must carefully choose *in vitro* cell models as well as clinical specimens to study the contribution of epigenetic modifications in pathology.

## Origin of risk and consequences on the assessment method

Logically, some risk is engraved in the tissue where disease starts; thus, in a first approach, this tissue should be the main location to study the epigenetic modifications that lead to altered cell functions preceding, as well as during, a disease. Increasing evidence shows that risk for chronic diseases might be established from birth or by environmental impact during childhood (Joss-Moore et al., 2011; Teegarden et al., 2012). Epigenetic remodeling, a normal process during organ development, makes the epigenome particularly sensitive to environmental impact. Moreover, epigenetic modifications can be carried within chromatin through mitotic division, indicating that sustained epigenetic marks in tissues of disease onset are good candidates for risk assessment. However, while prognosis on disease progression or treatment outcomes of a clinically established disease can usually be measured directly from the disease site itself, as part of the treatment process via biopsy or surgery, in primary prevention invasive or remote tissue access would be an issue for the assessment of risk for a clinically undetectable disease.

**Figure 1:**
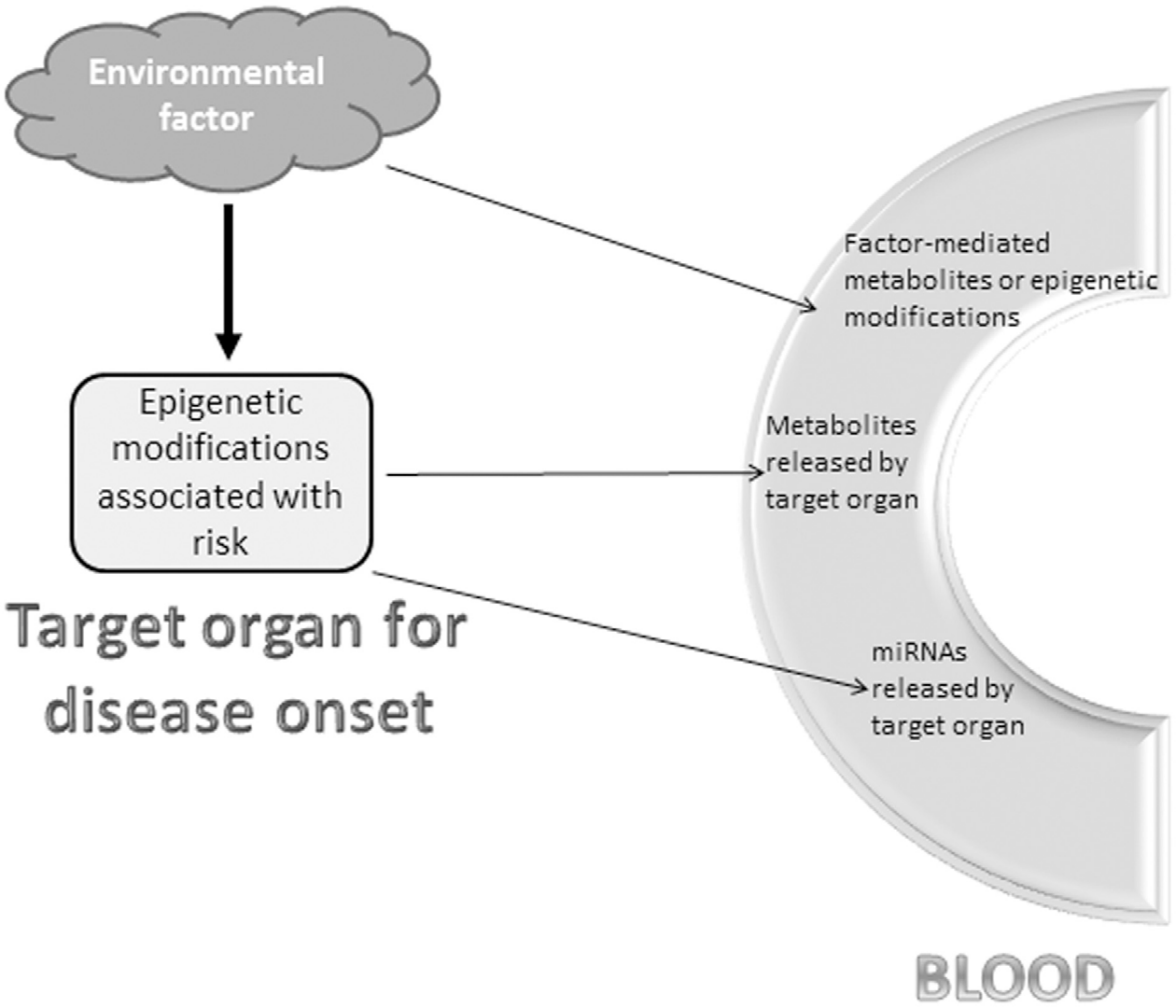
**Potential blood markers reflecting epigenetic changes of disease risk**. An environmental factor or risk exposure is a powerful means to modify the epigenome in certain organs and cell populations. Three non-exhaustive and non-exclusive options to detect the risk in the blood-stream include (i) blood metabolites or epigenetic modifications directly triggered by the environmental factor that also induced epigenetic modifications in the “at risk” or diseased organ, (ii) metabolites released into the blood-stream by the epigenetically-modified organ with increased risk for disease, and (iii) miRNAs synthesized upon environmental factor-mediated epigenetic modifications in the “at risk” or diseased organ and released into the blood-stream.

Whenever possible, a solution to the tissue sampling challenge is to initially carry out research on biopsy sections to decipher the local epigenome associated with a risk of specific disease; subsequently, we would identify blood markers that mirror the altered epigenetic status of the targeted organ. This situation might occur if the environmental impact on risk development introduced not only epigenetic changes in the organ site of the disease, but also direct modifications in the blood detectable by metabolomics or even epigenomics. Although epigenetic modifications in blood cells are primarily specific to this tissue and should be used with caution to prevent misleading risk assessment for another tissue, there might be instances for which these modifications could indeed be of use following sound confirmation studies. For example, similar epigenetic changes that target a set of genes involved in brain disorders like schizophrenia have been observed in both brain cells and peripheral lymphocytes (Guidotti et al., 2014). Blood markers might also be usable if the epigenetic modifications in the target organ have changed cellular activity in such a way that it is accompanied by the release of a specific set of blood metabolites or miRNAs (Figure 1). Indeed, blood-based detection of miRNAs has been used to reveal cancers (Liloglou et al., 2014) and the risk of aggressive neoplasia (Boeri et al., 2011).

Importantly, the modification of epigenetic marks by a risk or protection factor at any given time will depend on the epigenetic soil determined by the history of the individual in terms of ancestry and the life events that keep shaping the epigenome. Taking into account such epigenetic history is necessary to optimally ascertain the level of risk.

## The value of epigenetic history

An uncanny power of epigenetic marks lies in their propensity to carry an increased level of either protection or risk as set by the lifestyle of an individual's ancestor if these epigenetic modifications affected the gametes. This possibility has been reported for hypothalamic-pituitary-adrenal axis dysregulation and altered response to stress in offspring following stress periods in male rats induced before breeding. In this study, changes in the level of gene transcripts, notably of miRNAs, in brain regions that control stress were suggested to be evidence of epigenetic modifications of these genes in offspring (Rodgers et al., 2013). Moreover, the mother's lifestyle (e.g., nutrition, stress) during pregnancy also influences the offspring's epigenome (Vanhees et al., 2014). How long the impact has to be present to permanently engrave the genome is unclear. Nonetheless, it is undeniable that the epigenetic soil created by ancestry determines future epigenetic responses to environmental factors (Joss-Moore et al., 2011) and behaviors. In light of this growing evidence, I would argue that understanding the epigenetic soil should also be considered an integral part of risk assessment methods for individuals.

The complexity of the epigenetic code also implies its power and utility for risk assessment; it might permit the measurement of epigenetic marks of different strengths (e.g., temporary, stable) and thus, provide a means by which we can determine the origin of risk or whether an individual may react to a given environmental factor. This reasoning is not a futuristic view of medicine through which individuals would be aware of their level of protection against a particular disease, but rather follows from growing evidence that epigenetics will soon become an extremely precise tool for improving wellness.

However, the efficiency of risk assessment using epigenetic screens will be low unless experiments are carefully designed. For instance, comprehensive mapping of epigenetic modification sites will not help us understand disease risk unless normal variation at epigenetic sites is also investigated, taking into account ethnicity/race and even sociocultural background. Finally, the necessity to compare results on potential biomarkers of risk via meta-analyses is rendered difficult because of the plethora of methods used in epigenetics. Current and future databases that concentrate epigenetic data should use researcher-agreed upon standards, including clearly documented information on the means by which data were acquired.

## Conclusion

Risk assessment in healthcare remains a coveted yet unachieved goal. Our understanding of epigenetic marks offers a unique potential to personalize risk assessment and individualize treatment of diseases that rely on gene expression control for their onset and progression.

The care for most chronic diseases could be significantly improved by focusing on epigenetics in research, though we should not pursue epigenetic data for risk assessment without initiating discussions on the ethical impact of such endeavors. Through this statement, I wish to spur our thinking about the consequences of collecting potentially deep knowledge of a person's epigenome. For individuals who are clinically ill, epigenetic information is emerging as undoubtedly useful for treatment decisions. Once risk assessment pertains to the prevention of disease onset, epigenetic information will also come from disease-free individuals, concomitantly providing a mine of data on their ancestry and lifestyle. In addition to the protection of epigenetic data, risk assessment should be accompanied with a plan to reduce the risk of disease onset in agreement with the World Health Organization best practices for screening. Also, if samples from healthy individuals were only collected for epigenetic research purposes, it would seem logical that, as new risk assessment tools become available, research subjects themselves are given the possibility, if they wish, to access and potentially gain benefit from this information. This option would require that individuals participating in risk assessment be given the choice to be contacted in case new information gathered from the research becomes of use to them. In other words, more than ever before, risk assessment based on epigenetic history should embrace a relation of trust between scientists, (potential) patients and healthcare providers.

### Conflict of interest statement

The author declares that the research was conducted in the absence of any commercial or financial relationships that could be construed as a potential conflict of interest.
